# Gene and protein expression and cellular localisation of cytochrome P450 enzymes of the 1A, 2A, 2C, 2D and 2E subfamilies in equine intestine and liver

**DOI:** 10.1186/s13028-014-0069-8

**Published:** 2014-10-08

**Authors:** Eva Tydén, Hans Tjälve, Pia Larsson

**Affiliations:** Department of Biomedical Sciences and Veterinary Public Health, Division of Pathology, Pharmacology and Toxicology, Swedish University of Agricultural Sciences, S-750 07 Uppsala, Sweden

**Keywords:** CYP, Horse, Gene expression, Protein expression, Cellular localisation, Liver, Intestine

## Abstract

**Background:**

Among the cytochrome P450 enzymes (CYP), families 1–3 constitute almost half of total CYPs in mammals and play a central role in metabolism of a wide range of pharmaceuticals. This study investigated gene and protein expression and cellular localisation of CYP1A, CYP2A, CYP2C, CYP2D and CYP2E in equine intestine and liver. Real-time polymerase chain reaction (RT-PCR) was used to analyse gene expression, western blot to examine protein expression and immunohistochemical analyses to investigate cellular localisation.

**Results:**

CYP1A and CYP2C were the CYPs with the highest gene expression in the intestine and also showed considerable gene expression in the liver. CYP2E and CYP2A showed the highest gene expression in the liver. CYP2E showed moderate intestinal gene expression, whereas that of CYP2A was very low or undetectable. For CYP2D, rather low gene expression levels were found in both intestine and the liver. In the intestine, CYP gene expression levels, except for CYP2E, exhibited patterns resembling those of the proteins, indicating that intestinal protein expression of these CYPs is regulated at the transcriptional level. For CYP2E, the results showed that the intestinal gene expression did not correlate to any visible protein expression, indicating that intestinal protein expression of this CYP is regulated at the post-transcriptional level. Immunostaining of intestine tissue samples showed preferential CYP staining in enterocytes at the tips of intestinal villi in the small intestine. In the liver, all CYPs showed preferential localisation in the centrilobular hepatocytes.

**Conclusions:**

Overall, different gene expression profiles were displayed by the CYPs examined in equine intestine and liver. The CYPs present in the intestine may act in concert with those in the liver to affect the oral bioavailability and therapeutic efficiency of substrate drugs. In addition, they may play a role in first-pass metabolism of feed constituents and of herbal supplements used in equine practice.

## Background

Among the cytochrome P450 enzymes (CYP), families 1–3 constitute almost half of total CYPs in mammals. These are generally designated xenobiotic-metabolising enzymes and play a central role in metabolism of a wide range of xenobiotics [1]. The xenobiotic-metabolising CYPs are mainly expressed in the liver, but several are also expressed in extrahepatic tissues, particularly in organs in direct contact with xenobiotics, such as the intestines and the respiratory tissues [2,3]. Studies in different species have shown that each tissue may have its unique CYP expression pattern [2,3].

To date, three equine CYPs within families 1–3 have been cloned and functionally characterised. These are CYP2D50 [4], CYP2C92 [5] and CYP3A96 [6]. A substantial difference has been reported in the metabolic capacity of these equine CYPs compared with the human orthologs. For example, the metabolic capacity of CYP2D50, CYP2C92 and CYP3A96 is about 20- to 180-fold lower with regard to dextromethon-O-demethylation, diclofenac-oxidation and testosterone-6β-hydroxylation, respectively, than that of the human orthologs, CYP2D6, CYP2C9 and CYP3A4 [4-6].

There has been a continual increase in the volume of sales of drugs used in equine practice [7] and many drugs used in equine therapy are substrates for CYPs. There is also an increasing retail market for herbal supplements for horses [8]. However, little is known about drug-drug interactions and drug-herb interactions in the horse. Due to differences in expression level and substrate specificity of the CYP enzymes, it is difficult to extrapolate findings on the pharmacokinetics and pharmacodynamics of drugs between species [9,10]. Thus there is clearly a need for increased knowledge of the expression pattern and metabolic capacity of xenobiotic-metabolising CYP enzymes in the horse.

Previous studies by our group have examined expression of isoenzymes belonging to the CYP3A subfamily in the equine intestine and liver [11,12]. In the intestinal mucosa, CYP3A is considered to play an important role for the oral availability of substrate drugs and other xenobiotics, by sequential CYP3A-mediated metabolism in the intestine and liver [12,13]. However, studies in man and rodents have shown that, in addition to the CYP3A subfamily, other CYP subfamilies are also present in the intestinal mucosa and may play a role in modulating drug and xenobiotic bioavailability [13,14].

In the present study, we examined gene and protein expression and cellular localisation of CYPs of the 1A, 2A, 2C, 2D and 2E subfamilies in the intestine and liver of the horse. Gene expression was examined using quantitative real-time reverse transcriptase polymerase chain rraction (RT-qPCR). For the subfamilies CYP2C and CYP2D primers were designed based on conserved gene regions, thus enabling amplification of all isoenzymes belonging to these subfamilies. For the subfamilies CYP1A, CYP2A and CYP2E, primers were designed based on a single sequence. The reason for this was that these were the only sequences identified for these equine subfamilies at the time when this study was performed. Protein expression was examined by western blot. Immunohistochemical analysis was used to examine the cellular localisation of the CYPs in the intestine and liver of the horse.

## Methods

### Tissue sample collection

Tissues from healthy Swedish standard-bred trotters (n =8) were collected at a local slaughterhouse. The tissues were from 5- to 18-year-old mares and geldings. In accordance with Swedish National Food Administration regulations, the horses were examined by a veterinarian before slaughter. In addition, the passports of the horses were checked to ensure that all horses in the study were free of medication during the stipulated period prior to slaughter. Samples were collected from the liver and various parts of the intestines (Table 1), and tissue preparation was performed as described in [15]. In brief, for RNA analysis the mucosal surface was scraped smoothly with a scalpel, and samples were collected in Rnase-free plastic tubes and snap-frozen in liquid nitrogen. Liver tissue was treated in a similar way. Samples for protein expression were collected as described for RNA samples and transported to the laboratory in 0.9% NaCl at 4°C. Samples for immunohistochemical analysis (0.5 cm × 0.5 cm) were transported to the laboratory in 0.9% NaCl at 4°C and thereafter fixed in 4% paraformaldehyde.

**Table 1:** **Sampling sites along the intestinal tract of the horse**

**Tissue (code)**	**Position**
Duodenum (D1)	Immediately aboralaboral to the pyloric sphincter *(Pars prima duodeni)*
Duodenum (D2)	0.5 m aboral to the pyloric sphincter (*Pars secunda duodeni)*
Duodenum (D3)	1 m aboral to the pyloric sphincter *(Pars tertia duodeni)*
Jejunum (J1)	2.5 m aboral to the pyloric sphincter
Jejunum (J2)	5.5 m aboral to the pyloric sphincter
Jejunum (J3)	8.5 m aboral to the pyloric sphincter
Jejunum (J4)	13.5 m aboral to the pyloric sphincter
Ileum (I)	immediately anterior to the ileo-caecal orifice
Caecum (Ca)	Mid-part of the parietal surface of the *Corpus caeci*
Colon (Co)	At the origin of the small colon, aboral to the right dorsal colon

### Isolation of RNA

Total RNA was prepared using the NucleoSpin RNA II kit containing deoxyribonuclease I (DNase I) (Macherey-Nagel, Düren, Germany) according to the manufacturer’s instructions. The purity and integrity of the RNA were checked as described previously [16]. In brief, prior to real-time RT-PCR analysis the integrity of RNA was verified by examining ribosomal RNA 28 S and 18 S on 1% agarose gel containing 18% formaldehyde. Only RNA samples with a 260/280 nm ratio exceeding 1.8 were selected for real-time PCR. The exact amount of RNA was quantified using the RNA-specific Quant-iT RiboGreen protocol (Molecular Probes, Eugene, OR, USA) and a microplate reader (Wallac 1420 VICTOR^2^_TM_, software version 2.0, Turku, Finland).

### CYP gene expression

The gene sequences of the CYP2D subfamily were predicted by NCBI Map Viewer according to Yasukochi *et al*. [17]. The genes of the CYP2C subfamily were derived from drnelson homepage [18]. All other CYP genes were derived from the NCBI genome database (http://www.ncbi.nlm.nih.gov/). Primers for the genes of the CYP2C and CYP2D subfamilies were designed based on conserved regions, thus enabling amplification of all isoenzymes belonging to the same subfamily (Table 2). Primers for CYP1A, CYP2A and CYP2E were designed based on one sequence (Table 2). The primers were designed in Primer 3 (v. 0.4.0) [19] and synthesised by CyberGene AB, Stockholm, Sweden. Gene expression was examined by quantitative reverse transcriptase-polymerase chain reaction (RT-qPCR) using Rotor-Gene 3000 (Corbett Research, Mortlake, Australia) by applying a one-step QuantiTect®SYBR®green RT-PCR kit (Qiagen, Inc., Valencia, CA, USA) according to the manufacturer’s recommendations. The primer concentration was 0.4 μM and 300 ng total RNA were used. Copy number was determined using a standard curve derived from serial dilutions of known concentrations of the purified PCR products in the customary way. Copy number was calculated using the following formula: molecules μL^−1^ = (A × 6.022 × 10^23^) (660 × B)^−1^, where A is the concentration of the PCR product (g μL^−1^), B is the plasmid length, 6.022 × 10^23^ is Avogadro’s number, and 660 is the average molecular weight of one base pair. The amplification efficiency was calculated as (10^(−1/slope)^ − 1) × 100. The terminology used was in accordance with Bustin *et al*. [20]. The standard curve was created from purified PCR product stock solution that was serially diluted to give: 2×10^7^, 2×10^6^, 2×10^5^, 2×10^4^, 2×10^3^ copies μL^−1^. This was repeated several times to test the efficacy and reproducibility of the qPCR. Standard curves were created automatically and accepted when the slope was on average −3.69 ± 0.05 (97% efficacy) and the correlation coefficient (r^2^) was 1.00. The specificity of the PCR products obtained was checked in connection with each real-time RT-PCR run, using melt curve analysis. In addition, the PCR products were validated by checking the specificity and size on 1% agarose gel. The PCR products generated were sequenced at the Centre for Genomic Research, Karolinska Institute, Stockholm, Sweden.

**Table 2:** **Nucleotide sequences of primers**

**Name**	**Accession number**	**Sequence (5'-3')**	**Fragment length (bp)**
CYP1A	EU220011.1	F- CCGTTATCTGCCCAACTCTG	199
		R- CTTCTCGTCTGACAGCTGGA	
CYP2A	EU286274.1	F- GGGAACCGCTTTGACTATGA	199
		R- GCTCCACCTTCTTGGCTATG	
CYP2C	XM_001501993.1;XM_001502030.1 XM_001502107.2; XM_001502162.1 XM_001502179.2; XM_001500745.1 XM_001502230.1; XM_001502256.2 NM_001101652.1	F- AGCAATGGAAAGAGGTGGAA	201
		R- AATGGAGCAGATCACATTGC	
CYP2D	XM_001502856.1; XM_001916743.2	F- CATCTTCCTGCTCCTGGT	177
	XM_001502900.2; XM_001502807.2 XM_001917460.1; EU190996.1	R- AGCTGCAGGCTGAACAC	
CYP2E	EU232117.1	F- ACTATGGGATGGGGAAGCAG	202
		R- AAGTGCATCAGCCTCTGACA	

### CYP protein expression

Preparation of microsomes and determination of protein concentration were performed as described previously [15]. In brief, tissues were transported from the slaughterhouse in 0.9% NaCl at 4°C and within 40 min the samples (30 g) were homogenised in 10 mL cold 0.01 M KH_2_PO_4_/0.32 M KCl buffer, pH 7.4, containing 20% glycerol (vol/vol) and Complete EDTA-free Protease Inhibitor Cocktail Tablets (Roche Diagnostics GmbH, Mannheim, Germany). This was followed by three centrifugation steps; one at 10 000 × g for 20 min and two at 105 000 × g for 60 min. Aliquots of 50 μg of microsomal protein were separated on 10% Tris–HCl polyacrylamide gels under reducing conditions, as described previously [15]. Separated proteins were electroblotted to nitrocellulose membranes and transfer of proteins was confirmed by staining with Ponceau S (Sigma-Aldrich, St. Louis, MO, USA). Membranes were blocked in 10% dry milk and incubated with primary antibodies (Table 3), diluted in phosphate-buffered saline (PBS) containing 0.1% Tween-20 (PBST). For CYP1A, CYP2A, CYP2D and CYP2E, a secondary goat-anti-rabbit antibody (AQ132P, Chemicon International, Temecula, CA, USA) conjugated with horseradish peroxidase (HRP) was used, diluted 1:50 000 in 2% advanced blocking buffer (GE Healthcare, Uppsala, Sweden). HRP was detected by Enhanced Chemiluminescence (ECL) western blot Detection reagents (GE Healthcare, Uppsala, Sweden). For CYP2C, a secondary mouse-anti-sheep antibody (213-032-177, Jackson ImmunoResearch Laboratories, West Grove, PA, USA) was used and diluted 1:1000 as described above. The ECL reagents were used according to the manufacturer’s instructions. The Chemi-Doc Gel Quantification System (Bio-Rad, Hercules, CA, USA) and Quantity-One software were applied to detect the intensity of the bands. The blotting membranes were stripped using 65 mM Tris–HCl, 100 mM 2-mercaptoethanol, 2% SDS for 5 min at 55–60°C, rinsed three times with PBST and thereafter incubated with the next primary and secondary antibodies.

**Table 3:** **Polyclonal antibodies used in immunohistochemical (IHC) and western blot (WB) analyses**

**CYP-enzyme**	**Primary anti-body**	**Company**	**Dilution**
CYP1A	Polyclonal rabbit-anti-rat (AB1247) CYP1A1	Chemicon International, Temecula, CA, USA	WB: 1:2000
			IHC: 1:1000
CYP2A	Polyclonal rabbit-anti-human (CR3260) CP2A6	Enzo Life Sciences, Farmingdale, NY, USA	WB: 1:2000
			IHC 1: 1000
CYP2C	Polyclonal sheep-anti-human/rat (CR3285) CYP2C8; 2C9; 2C19; rat 2C12	Enzo Life Sciences, Farmingdale, NY, USA	WB: 1:1000
			IHC:1: 4000
CYP2D	Polyclonal rabbit-anti-rat (CR3210) CYP2D1	Enzo Life Sciences, Farmingdale, NY, USA	WB: 1:1000
			IHC: 1:2000
CYP2E	Polyclonal rabbit-anti-human/rat (CR3271) CYP2E1	BIOMOL International, LP, USA	WB: 1:1000
			IHC: 1:2000

### Immunohistochemical analysis

Pieces of intestine and liver tissue were fixed in formaldehyde, dehydrated and embedded in paraffin. From these, 5 μm tissue sections were taken and deparaffinised, hydrated and rinsed in phosphate-buffered saline (PBS). The rinsing procedure, involving two rinsing intervals in PBS for 10 min, was repeated after each of the steps described below. Endogenous peroxidase activity was blocked with 1.6% H_2_O_2_ in PBS, followed by incubation for 1 hour in PBS with 10% normal goat serum (for CYP1A, CYP 2A, CYP 2D and CYP 2E) or normal rabbit serum (for CYP2C). Endogenous avidin and biotin were blocked using the Avidin/Biotin Blocking kit (Vector Laboratories Inc., Burlingame, CA, USA). Thereafter, the tissue sections were incubated overnight at 4°C with the primary antibodies diluted in PBS as shown in Table 3. The secondary antibodies (goat-anti-rabbit or rabbit-anti-sheep; Vector Laboratories Inc., Burlingame, CA, USA) were used at 1:200 dilution and applied for 1 h at room temperature. The antigen-antibody complex was conjugated with avidin-biotin peroxidase using the ABC Vectastatin kit and then visualised with DAB staining, according to the supplier’s recommendations (Dako, Glostrup, Denmark). Finally, the sections were counterstained with haematoxylin (VWR International AB, Stockholm, Sweden). Negative controls were run in parallel with non-immune rabbit (CYP1A, 2A, 2E, 2D) or sheep (CYP2C) IgG (Abcam, Cambridge, UK) in amounts equivalent to those used for the antibodies against the CYP enzymes.

### Statistical analysis

All statistical analyses were performed using Minitab® software, version 15 (Minitab Inc., State College, PA, USA). Levene’s test was used to test whether the data followed a normal distribution. A paired *t*-test or a one-way analysis of variance (ANOVA) was performed to test for differences between groups. Dunnett’s test was used as a post hoc test in combination with ANOVA. Differences were considered significant at *P* < 0.05.

## Results

### CYP gene expression

For CYP2A, the gene expression level was very low or even undetectable in all parts of the intestine, whereas gene expression in the liver was high for this CYP (Table 4). For the other CYPs, the CYP expression levels were higher in all parts of the small intestine (duodenum, jejunum and ileum) than in the large intestine (caecum and colon). CYP1A and CYP2C showed the highest intestinal gene expression. Both these CYPs also showed considerable hepatic gene expression levels. CYP2E showed moderate intestinal gene expression and high hepatic gene expression. For CYP2D, the gene expression levels were rather low in both intestine and liver.

**Table 4:** Gene expression of CYP subfamilies in equine intestine and liver*

**Tissue**	**CYP1A**	**CYP2A**	**CYP2C**	**CYP2D**	**CYP2E**
D1	630 ± 130	4 ± 2	1360 ± 690	20 ± 20	40 ± 20
D2	1100 ± 240	5 ± 4	2270 ± 1150	30 ± 20	90 ± 100
D3	730 ± 130	3 ± 1	2200 ± 1290	30 ± 20	140 ± 170
J1	6600 ± 70	3 ± 2	2010 ± 1330	30 ± 20	130 ± 120
J2	1000 ± 10	2 ± 1	1680 ± 110	40 ± 20	190 ± 150
J3	1070 ± 20	2 ± 3	1380 ± 860	40 ± 20	160 ± 160
J4	660 ± 100	2 ± 1	1240 ± 510	30 ± 20	120 ± 130
I	600 ± 110	2 ± 1	810 ± 580	10 ± 8	140 ± 130
Ca	20 ± 20	1 ± 2	190 ± 170	1 ± 2	10 ± 20
Co	60 ± 70	2 ± 2	100 ± 50	1 ± 2	30 ± 40
Liver	1400 ± 950	13400 ± 8600	3200 ± 200	130 ± 60	11400 ± 8100

Data given as absolute quantification in molecules/μL.

*For tissue codes, see Table 1.

Statistical analysis of the differences between the hepatic and small intestinal gene expression levels revealed that CYP2A and CYP2E had significantly higher hepatic than intestinal gene expression (*P* < 0.05). For CYP1A, CYP2C and CYP2D, there were no statistical differences between gene expression levels in the small intestine and liver.

### CYP protein expression

**Figure 1:**
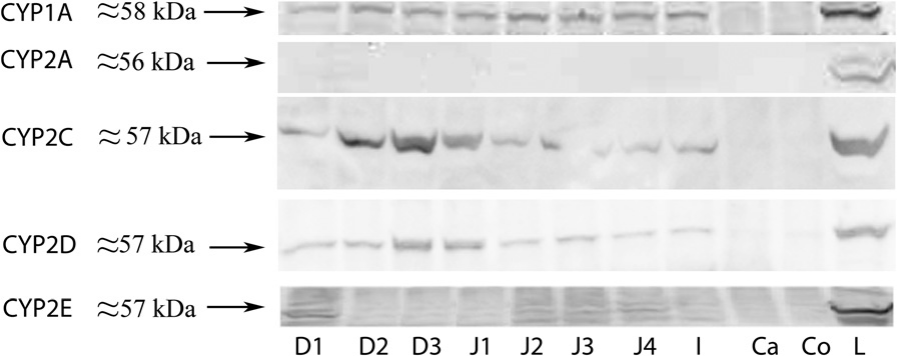
**Western blot.** Representative western blots of CYP1A, 2A, 2C, 2D and 2E in the intestine and the liver. D1-3; sections of duodenum, J1-4; sections of jejunum, I; ileum, Ca, Caecum, Co; colon, L, liver.

Representative western blots of microsomal proteins in the liver resulted in detection of immunopositive bands at 56–58 kDa for all CYPs (Figure 1). For CYP1A, CYP2C and CYP2D, immunopositive bands of this size were seen in the small intestine, whereas in the large intestine such bands were either absent or barely detectable (Figure 1). For CYP2E, it was not possible to clearly detect immunopositive bands at 56–58 kDa in any part of the intestine. For CYP2A, the western blots showed a complete lack of intestinal immunostaining.

### Immunohistochemistry

**Figure 2:**
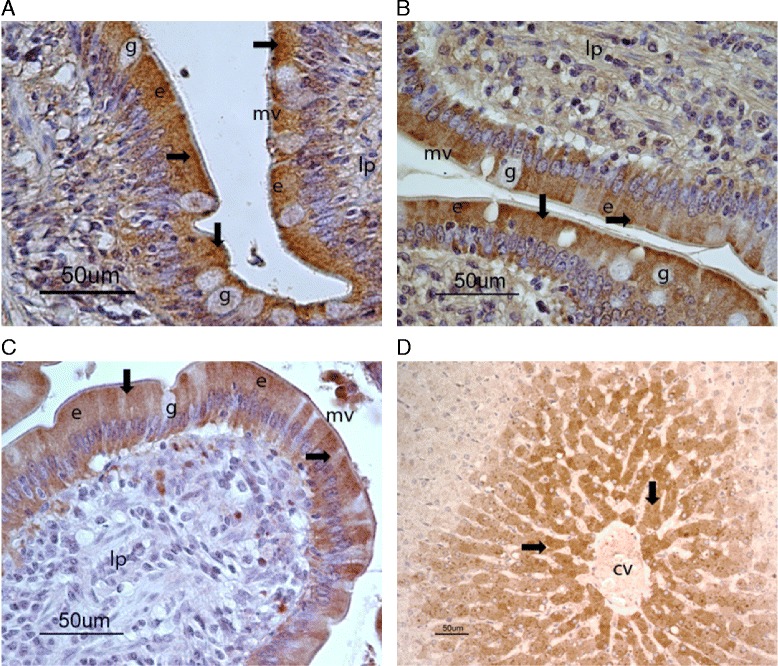
**Cellular CYP-localisation in the intestines and the liver.** Immunohistochemical staining of CYP1A **(A)**, CYP2C **(B)**, CYP2D **(C)** and CYP2E **(D)** enzymes in the duodenum **(C)**, jejunum **(A,B)**, and liver **(D)** in horse. The immunoreactivity of the CYP antibodies, indicated by arrows, are shown in brown colour. The nuclei of the cells blue colored with Heamatoxylin are easily seen. Legends: cv, central vein; e, enterocyte; g, goblet cell; lp, lamina propia; mv, microvilli of enterocytes. Magnifications are indicated by bars.

For CYP1A, CYP2C and CYP2D, the immunohistochemical analysis showed marked immunostaining of the enterocytes of the small intestine (duodenum, jejunum and ileum) and weak immunostaining of the enterocytes of the large intestine (caecum and colon). There was no detectable intestinal immunostaining for CYP2A and CYP2E. The immunostaining observed for CYP1A, CYP2C and CYP2D was strongest in enterocytes at the tips of intestinal villi in the small intestine, with preferential localisation in the outer parts of the cytoplasm of the enterocytes, towards the intestinal lumen (Figure 2A-C). Goblet cells did not stain and there was no immunostaining in the lamina propria or muscularis mucosae. In the liver, immunostaining was seen for all CYPs, with the strongest staining in hepatocytes in central parts of the hepatic lobuli (Figure 2D).

## Discussion

This study showed that gene expression levels in equine intestine and liver usually display particular patterns for the different CYP isoforms. In the intestine, gene expression was high for CYP2C and CYP1A, low for CYP2E and CYP2D and almost undetectable for CYP2A. In the liver, gene expression was high for CYP2A and CYP2E, moderate for CYP2C and CYP1A and low for CYP2D. The intestinal gene expression of the CYPs examined in the present study was higher in the small intestine than in the large intestine. This was also observed for CYP3A in our previous studies in the horse [11,12]. Similar distribution patterns in gene expression of CYPs along the gastrointesinal tract have been observed in other species [13,21,22].

The present study demonstrated high levels of gene expression for CYP1A and CYP2C in the equine small intestine. As mentioned, our previous studies have shown that CYP3A is also highly expressed in equine intestine [11,12]. CYP3A and CYP2C represent the major CYPs expressed in human intestinal mucosa, but this is not generally the case for CYP1A [13,23]. It has been shown that CYP1A1 expression in the human intestine is highly inducible, whereas there is consitutive expression of CYP3A and CYP2C [23]. The high expression of CYP1A observed here in the equine intestine can be related to the presence of CYP1A-inducing components in the diet of the horse. These differences in CYP isoform expression in the intestine must be considered when extrapolating data between species.

The CYP2D gene and protein expression was found to be low in both the liver and intestine of the horse. Yasukoch & Satta [17] examined the evolution of the CYP2D gene cluster and found that the number of members within the CYP2D subfamily varies between species. For example, primates have two to three CYP2D genes, whereas rodents, rabbits and horses have seven, five and six CYP2D genes, respectively. It has been suggested that the expansion of members within the CYP2D subfamily in herbivores might be related to the fact that several plant toxins are substrates for the CYP2D enzyme [17,24]. It is interesting that the expansion of the CYP2D subfamily in the equine genome was not accompanied by prominent gene or protein expression in the equine liver and intestine in the present study. The human CYP2D6 is known to be a polymorphic CYP isoenzyme with highly variable gene expression [25].

CYP2A and CYP2E were highly expressed in the equine liver, but both had very low gene expression levels in the intestine. This is consistent with reported levels in humans, where CYP2E1 is highly expressed in the liver, but only weakly expressed in the intestine for review see [26]. Bièche *et al.* [27] have shown high hepatic gene expression and very low intestinal gene expression for the three members of the human CYP2A subfamily.

Our present and prevous studies have shown that the gene expression levels of CYP1A, CYP2C and CYP3A in the equine small intestine were comparable to those in the liver. These results differ from observations in humans and dogs, in which the CYP expression levels in the liver are generally much higher compared than those in the small intestine [27,28]. It is possible that the high levels of CYPs in the equine intestine relate to the fact that the horse is a herbivorous species, which means that the diet may contain various CYP-inducing substrates, including phytonutrients and phytotoxicants. Consequently, during their evolution horses may have developed a more effective intestinal CYP system than omnivores or carnivores such as humans and dogs.

In the equine intestine and the liver, the CYP gene expression levels, except for CYP2E, exhibited expression patterns resembling those of the proteins, as shown by western blot analysis (Figure 1). This confirms findings in other species indicating that CYPs in general are regulated at the transcriptional level [29,30]. As regards CYP2E, our results showed that the intestinal gene expression detected in the PCRs did not correlate to any clearly detectable CYP2E protein expression in the western blots. This indicates that the protein expression of CYP2E is regulated at the post-transcriptional level. Similarly, studies with human liver biopsies have shown that the mRNA levels for CYP2E1 do not correlate to the CYP2E1 protein levels [31]. In addition, studies by Rodriguez-Antona *et al.* [32] have shown that there is no significant correlation between CYP2E mRNA expression and CYP2E-related metabolic activity in human liver samples.

Our immunohistochemical analyses showed that for the CYPs for which intestinal immunostaining was observed (CYP1A, CYP2C and CYP2D), there was preferential localisation of the staining in the enterocytes at the tips of the villi in the small intestine. We have previously shown that this staining pattern also applies for CYP3A in the equine intestine [11]. Similar findings have been made in other species [33,34]. In the liver, marked immunostaining was seen for all CYPs, with the strongest staining in hepatocytes in central parts of the hepatic lobuli. These results also corroborate those in other species (for review see [35]).

Many CYPs have been shown to be metabolically active in horses and, on the whole, oxidative drug metabolism appears more extensive in horses than in man [36]. Many drugs used in equine therapy, such as quinolones [37], dexamethasone [38], ivermectin [39,40], benzimidazoles [41,42], ketamine [43], meloxicam [44], omeprazole [45], phenylbutazone [46], praziquantel [47,48] and pyrantel [47], are substrates for the CYP enzymes. Several herbal supplements used in equine practice have also been reported to be CYP substrates. Examples are quercetin, the active component in devil’s claw root [49]; ginsengoides, the active components in ginseng [49]; and silymarin, the active component in meadowsweet [50]. It is also known that CYP-inducible components, such as flavonoids [51], are present in the normal diet of the horse, which may indicate that the equine CYPs have been strongly subjected to positive selection. It is apparent that there is a need for further studies on the expression patterns, metabolic capacities and inducibility of CYP enzymes in the horse.

## Conclusions

This study demonstrated differing gene and protein expression profiles of the five CYPs studied in equine intestine and liver. The CYPs present in the intestine may act in concert with those in the liver to affect the oral bioavailability and therapeutic efficiency of substrate drugs. In addition, they may play a role in first-pass metabolism of equine feed constituents and of herbal supplements used in equine practice.
